# A reference genome for *Trichogramma kaykai*: a tiny desert-dwelling parasitoid wasp with competing sex-ratio distorters

**DOI:** 10.1093/g3journal/jkaf129

**Published:** 2025-06-06

**Authors:** Jack A Culotta, Amelia R I Lindsey

**Affiliations:** Department of Entomology, University of Minnesota Twin Cities, 1980 Folwell Avenue, Saint Paul, MN 51108, USA; Department of Entomology, University of Minnesota Twin Cities, 1980 Folwell Avenue, Saint Paul, MN 51108, USA

**Keywords:** *Wolbachia*, sex ratio, selfish genetic element, symbiosis, B-chromosome, parthenogenesis, genome assembly

## Abstract

The tiny parasitoid wasp *Trichogramma kaykai* inhabits the Mojave Desert of the southwest United States. Populations of this tiny insect variably host up to 2 different sex-distorting genetic elements: (1) the endosymbiotic bacterium *Wolbachia* which induces the parthenogenetic reproduction of females, and (2) a B-chromosome, “Paternal Sex Ratio” (PSR), which converts would-be female offspring to PSR-transmitting males. We report here the genome of a *Wolbachia*-infected *T. kaykai* isofemale colony KSX58. Using Oxford Nanopore sequencing, we produced a final genome assembly of 205 Mbp with 34× coverage, consisting of 154 contigs with an N50 of 2.2 Mbp. The assembly is quite complete, with 92.67% complete Hymenoptera BUSCOs recovered: a very high score for Trichogrammatids that have been previously characterized for having high levels of core gene losses. We also report a complete mitochondrial genome for *T. kaykai*, and an assembly of the associated *Wolbachia*, strain *w*Tkk. Finally, we identified copies of the parthenogenesis-inducing (PI) genes *pifA* and *pifB* in a remnant prophage region of the *w*Tkk genome and compared their evolution to *pifs* from a suite of other PI *Wolbachia*. The *T. kaykai* assembly is one of the highest quality genome assemblies for the genus to date and will serve as a great resource for understanding the evolution of sex and selfish genetic elements.

## Teaser

Reproduction in the parasitic wasp *Trichogramma kaykai* is determined by the presence of a bacterial symbiont that converts offspring to female (“parthenogenesis”), and a selfish male-making chromosome. This work presents sequencing, assembly, and analysis of the wasp’s genome, and that of its *Wolbachia* symbiont. Genes involved in parthenogenesis induction are identified and comparative analyses across *Trichogramma* reveal conservation within the genus. These genome resources will enhance our ability to study reproductive biology and support a foundational understanding of *Trichogramma*, which are applied globally to manage agricultural pests.

## Introduction

*Trichogramma* wasps (Hymenoptera: Trichogrammatidae) are some of the smallest animals on the planet (Polilov 2015). The genus contains more than 200 described species: all parasitoids that complete their development within the eggs of other insects (Pinto 2006; Burks *et al*. 2024). Trichogrammatid research has largely focused on (1) their application as biological control agents of insect pests (Knutson 1998; Cherif *et al*. 2021), (2) innovations associated with extreme miniaturization (Polilov 2012), and (3) sex allocation, especially due to relationships with sex-distorting elements (Stouthamer *et al*. 1990; Stouthamer and Kazmer 1994; Russell and Stouthamer 2010). The most common sex-ratio distorter is the intracellular, maternally transmitted bacterium *Wolbachia*, a common associate of many arthropods and nematodes (Kaur *et al*. 2021). In *Trichogramma*, most *Wolbachia* strains are “parthenogenesis-inducing” (PI), and enable the asexual reproduction of females (i.e. “thelytokous parthenogenesis”) (Stouthamer *et al*. 1990, 1993; Ma and Schwander 2017).

**Fig. 1.**
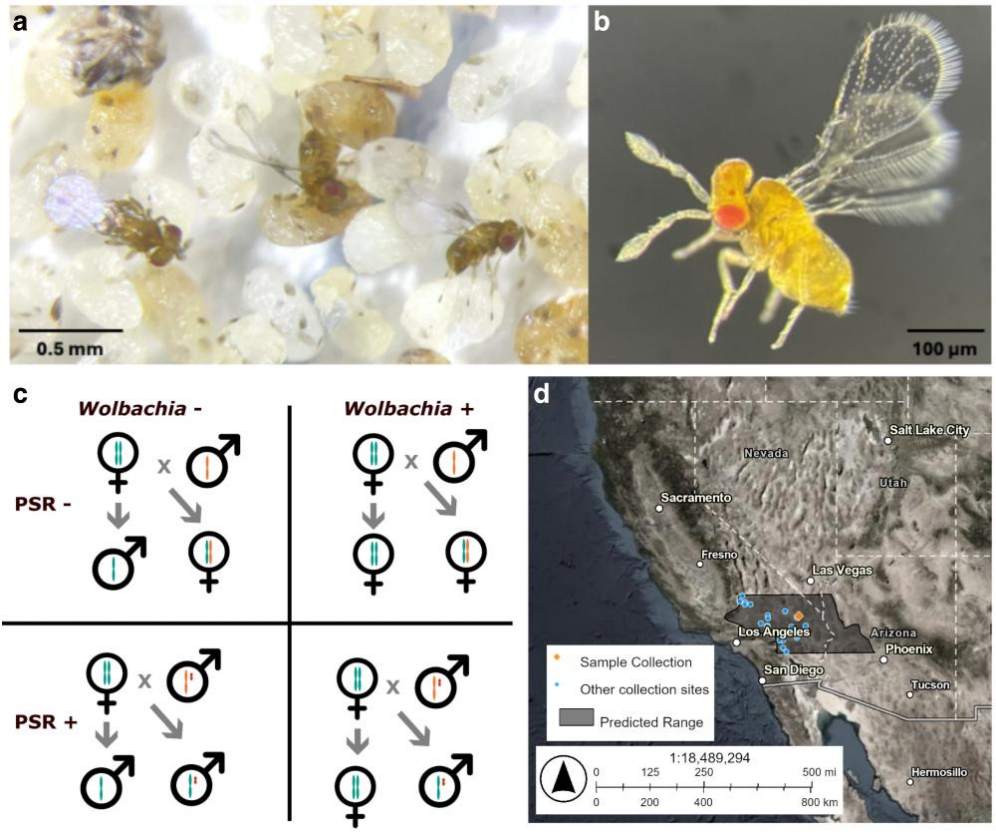
*Trichogramma kaykai* biology. a) Three *T. kaykai* females ovipositing into host moth eggs (*E. kuehniella*). b) An exemplary specimen of *T. kaykai* (female). c) Sex in *T. kaykai* is determined based on haplodiploidy, mediated by the presence or absence of *Wolbachia* (maternally transmitted) and the PSR chromosome (paternally transmitted). d) Range map of *T. kaykai.* The predicted geographic range is shaded. The sample collection site for KSX58 is represented by a diamond, and other known collection locations of *T. kaykai* are circles. Basemap data sources: Esri, Maxar, Earthstar Geographics, and the GIS User Community, Mono County, TomTom, Garmin, FAO, NOAA, USGS, EPA, USFWS.

To date, all instances of microbe-mediated PI are in animals with haplodiploid sex determination (Ma and Schwander 2017; Verhulst *et al*. 2023). Under haplodiploidy (and without PI-*Wolbachia*) males typically develop from unfertilized (i.e. haploid) eggs, and females are typically derived from fertilized, diploid, eggs (De La Filia *et al*. 2015). PI-*Wolbachia* diplodize the unfertilized eggs, resulting in a female (Stouthamer and Kazmer 1994). In *Trichogramma kaykai*, host to PI-*Wolbachia* (Fig. 1, a and b), a second sex-distorter is sometimes present: a supernumerary B-chromosome, “Paternal Sex Ratio” (PSR) (Stouthamer *et al*. 2001; van Vugt *et al*. 2003). PSR achieves the opposite outcome of *Wolbachia*'s PI: haploid males with PSR mate, and any fertilized eggs develop into more PSR-transmitting males (Van Vugt *et al*. 2009). PSR facilitates destruction of the paternal genome (except for itself), resulting in a haploid embryo (the maternal copy) and the untouched PSR chromosome. PSR is carried by ∼10% of males, though some populations of *T. kaykai* are PSR-free, and in others nearly a third of males carry this chromosome (Stouthamer *et al*. 2001). In populations where both *Wolbachia* and PSR are present, a curious pattern of reproduction is present: males are derived from fertilized eggs (with PSR-containing sperm), and females are derived from unfertilized eggs (with PI-*Wolbachia*) (Fig. 1c). Unlike many other PI-*Wolbachia* systems where PI is accompanied by a decay of sexual function (Gottlieb and Zchori-Fein 2001; Stouthamer and Mak 2002; Jeong and Stouthamer 2005; Stouthamer *et al*. 2010; Russell and Stouthamer 2011), *T. kaykai* are easily cured of their *Wolbachia* in the lab, and readily return to a fully functional sexual form (Hohmann and Luck 2000; Hohmann *et al*. 2001; Miura and Tagami 2004; Russell *et al*. 2016). The PSR chromosome ensures males and sexual reproduction are maintained.

As host to PI-*Wolbachia* and PSR, *T. kaykai* is a valuable model for understanding the evolution of sex ratios and interactions between selfish genetic elements. This species was described in 1997 (Pinto *et al*. 1997) and is native to the deserts of the Southwest United States (Fig. 1d). We report a reference genome for an isofemale colony of *T. kaykai* from the Mojave Desert, plus the genome of its PI-*Wolbachia* strain, *w*Tkk. To our knowledge, there are currently no *T. kaykai* PSR chromosomes in culture, but this reference genome will aid in future efforts to understand how this selfish element alters chromosome dynamics and sex ratios.

## Materials and methods

### Species origin and sampling strategy

Genome sequencing and assembly was performed for *T. kaykai* line “KSX58,” an isofemale laboratory culture. A single unmated *Wolbachia*-infected, thelytokous female was reared out of a parasitized *Apodemia mormo* egg collected off an *Eriogonum inflatum* stem and used to initiate an isofemale line. The founding female was collected in May 2010 in Kelso, CA, USA, by R. Stouthamer and J. Russell (Fig. 1d). The colony has since been maintained in 5 mL glass culture tubes stopped with cotton, and kept at 25°C with a 12:12 light:dark cycle. Wasps are hosted every 12 days on sterilized *Ephestia kuehniella* eggs adhered to cardstock alongside a streak of honey. *Wolbachia* infection status was confirmed by PCR with *Wolbachia* specific “Wspec” primers (Werren and Windsor 2000), and *Trichogramma* species was confirmed by molecular identification (Stouthamer *et al*. 1999), both as detailed previously (Lindsey and Stouthamer 2017). To collect wasps for DNA extraction, freshly emerged females were allowed to crawl up into a sterile tube attached to the colony culture vial. The pool of wasps was flash frozen in liquid nitrogen and stored at −80°C for further processing.

### Geographic range mapping

Locations of *T. kaykai* are centered around the Southern Mojave Desert (Fig. 1d) (Pinto *et al*. 1997; van Vugt *et al*. 2003, 2009; Tulgetske and Stouthamer 2012; Russell *et al*. 2016, 2018). The predicted northern and southern boundaries of this species' range were estimated from these observations. As it is assumed *T. kaykai* is restricted to desert habitat, the eastern and western borders of its range are indicated by the Southern Mojave Desert and Northern Sonoran Desert. The map was generated in ArcGIS Online (www.arcgis.com).

### Sequencing methods and sample preparation

DNA was extracted from 25 mg of whole insect tissues using the MagAttract High Molecular Weight kit (Qiagen), following manufacturer's instructions. The DNA was concentrated to 25 µL using Sergi Lab Supplies magnetic beads and went through the PacBio SRE kit to deplete fragments shorter than 10 kb. The sample was barcoded and library prepped with the ONT SQK-NBD114.24 kit. The libraries were sequenced on a P2 Solo instrument using PromethION 10.4.1 flow cells. Every 24 h the libraries were recovered and flow cells were flushed with nuclease (EXP-WSH004 kit) and reloaded.

### Nuclear genome assembly, curation, and quality control

Samples were originally basecalled within Minknow using “super accuracy” mode with 5mC_5hmC modified base calling. Reads were then re-basecalled with Dorado v.0.7.2 using basecall model dna_r10.4.1_e8.2_400bps_sup\@v5.0.0. Reads at least 5 kb in length were maintained, processed with “dorado correct,” and used for generating an assembly with Hifiasm v.0.19.9 and default parameters. Three rounds of scaffolding with gap filling were completed with ntLink v4.4.1 (Coombe *et al*. 2023). Purge_Dups v1.2.6 was used to remove duplicate haplotigs and overlaps (Guan *et al*. 2020). The genome was curated for contamination with FCS v0.5.4 (Astashyn *et al*. 2024), and cytoplasmic genomes were identified through a combination of FCS results, and tblastn results as implemented in Blobtools v.1.1.1 (Challis *et al*. 2020). K-mer analysis was performed with Merqury v1.3 (Rhie *et al*. 2020). Assemblies were assessed with Compleasm v.0.2.6 (Huang and Li 2023) with the hymenoptera lineage flag (‘-l hymenoptera’).

### Genomic methylation

Methylation and hydroxymethylation of genomic DNA at 5′ cytosines (5mC and 5hmC) in a cytosine-guanine dinucleotide (CpG) context were determined from the basecalling information stored in the unmapped modBAM files (Flack *et al*. 2024). These were aligned to the final assembly using Minimap v.2.17 (Li 2016), converted to bedMethyl format with Modkit v.0.4.1 (https://github.com/nanoporetech/modkit), and the 5mC and 5hmC percentages were calculated with an AWK script.

### *Trichogramma* phylogeny

A whole-genome phylogeny was reconstructed with SANS v.2.4_10, which uses a pangenomic approach to calculate splits in a phylogenetic tree (Rempel and Wittler 2021). SANS parameters included “–filter strict” with an output Newick tree file and 100 bootstrap replicates. Taxa included the available *Trichogramma* genomes (for *Trichogramma brassicae*, which is represented by 2 assemblies, only GCA_902806795.1 was used; Table 1), and an outgroup species from a closely related family (Cruaud *et al*. 2024), *Phymastichus coffea* (Hymenoptera: Eulophidae) GCF_024137745.1. Tree topology was configured in FigTree v.1.4.4 (https://github.com/rambaut/figtree/) and annotated in Inkscape (https://www.inkscape.org).

**Table 1.** *Trichogramma* genome assemblies.

Species	Accession	Size (bp)	Contig/scaffold count^a^	N50 (bp)^a^	Compleasm^b^	*Wolbachia* genome	Citation
*Trichogramma brassicae*	GCA_902806795.1	235,386,796	1,570 (C)	556,663	91.84% [S: 90.44%, D: 1.40%] F: 0.85%, M: 7.31%, *n* = 5991	No	Ferguson *et al*. (2020)
*Trichogramma brassicae*	GCA_030522885.1	203,810,232	87,792 (S)	18,131	86.78% [S: 85.93%, D: 0.85%] F: 4.26%, M: 8.90%, *n* = 5991	No	Guinet *et al*. (2023)
*Trichogramma dendrolimi*	GCA_034770305.1	215,209,100	316 (S)	1,412,680	89.30% [S: 85.16%, D: 3.14%] F: 0.63%, M: 11.07%, *n* = 5991	No	Zhang *et al*. (2023)
*Trichogramma evanescens*	GCA_902732785.1	213,671,129	146,286 (S)	38,173	89.63% [S: 88.48%, D: 1.15%] F: 2.69%, M: 7.64%, *n* = 5991	No	N/A
*Trichogramma pretiosum*	GCA_000599845.3	187,641,947	925 (S)	1,825,723	93.04% [S: 92.02%, D: 1.02%] F: 0.73%, M: 6.23%, *n* = 5991	*w*Tpre Lindsey *et al*. (2016)	Lindsey *et al*. (2018a)
*Trichogramma kaykai*	GCA_045785165.2	205,183,918	154 (C)	2,221,389	92.67% [S: 91.74%, D: 0.93%] F: 0.82%, M: 6.51%, *n* = 5991	*w*Tkk; This study	This study

^a^If assembly is comprised of scaffolded contigs, metrics reported are for scaffolds and an (S) is indicated in the count column. If there are only contiguous sequences, those metrics are reported and (C) is indicated in the count column.

^b^Standard BUSCO annotation: Complete BUSCOs (C) [Complete and single-copy BUSCOs (S), Complete and duplicated BUSCOs (D)], Fragmented BUSCOs (F), Missing BUSCOs (M), Total BUSCO groups searched (n). Hymenoptera dataset used for determining completeness.

### Repeat assembly techniques

We identified and masked repetitive sequences in each genome. First, a custom de novo repeat library was created with RepeatModeler v.2.0.5 (Flynn *et al*. 2020) with the -LTRStruct parameter included. Then this library was used to mask the genome with RepeatMasker v.4.1.1 (Tarailo-Graovac and Chen 2009) with the -s (sensitive mode) parameter included.

### Gene finding methods

To annotate the *T. kaykai* genome, a soft-masked genome was used for gene model prediction with Braker v.3.0.8 (Hoff *et al*. 2019; Brůna *et al*. 2021). The Braker pipeline was executed using Singularity with parameters to use Arthopoda protein data from OrthoDB v.11 (Kuznetsov *et al*. 2023), and to output a gff3 file. Summary statistics for the resulting gff3 file were computed with GAG v.2.0.1 (Geib *et al*. 2018).

### Synteny analysis

We identified conserved regions and mapped synteny between the *T. kaykai* and *T. pretiosum* genomes (Table 1) using the D-GENIES webtool (https://dgenies.toulouse.inra.fr/run) (Cabanettes and Klopp 2018) employing Minimap v.2.28 (Li 2016), the “many repeats” flag, and the “hide noise” option.

### Mitogenome

A single circular contig was identified as the mitochondrial genome based on GC content, size, and coverage. Mitogenome annotation was completed with MITOS2 v.2.1.9 (Bernt *et al*. 2013; Donath *et al*. 2019), and the circular mitogenome was started at Cox1 per convention with rearrangement in SnapGene v.7.2. MITOS2 parameters were the RefSeq63 Metazoa reference and the invertebrate mitochondrial translation code. Manual curation of the control region and inferences of gene structure were made based on comparisons to other *Trichogramma* mitochondrial genomes (Chen *et al*. 2018).

### *Wolbachia* strain *w*Tkk genome

Prior to scaffolding the nuclear *T. kaykai* assembly, 4 contigs were identified as a *Wolbachia* genome based on cumulative size and Blobtools results. Scaffolding was attempted with ntLink v4.4.1 (Coombe *et al*. 2023), but did not yield a circularized genome. Genome completeness was analyzed against the rickettsiales_odb10 database with Compleasm v.0.2.6 (Huang and Li 2023). Prophage regions and mobile elements were identified with VirSorter2 v.2.2.4 (Guo *et al*. 2021) and mobileOG-db v.1.0.1 (Brown *et al*. 2022), implemented in proksee (Grant *et al*. 2023) (https://proksee.ca/) with default parameters. To identify putative parthenogenesis-inducing genes (*pifs*) (Fricke and Lindsey 2024), we leveraged annotation and orthology data generated by Prokka v.1.14.6 (Seemann 2014) and OrthoFinder v.2.5.4 (Emms and Kelly 2019), implemented in the *Wolbachia* Phylogeny Pipeline (WHOP; https://github.com/gerthmicha/WHOP). Phylogenetic analysis was performed based on the clustering results from WHOP/OrthoFinder results. Single-copy orthologs were aligned with MAFFT L-INS-i v.7.487 (Katoh and Standley 2013), recombining genes were eliminated with PhiPack v.1.1 (Bruen and Bruen 2005), and alignments were concatenated for phylogenetic reconstruction in IQtree v.2.2.3 (Nguyen *et al*. 2015), run with model optimization and 1,000 ultrafast bootstrap replicates. Heatmaps of protein divergence were generated in R version 4.4.1 using percent identity values from Clustalo v.1.2.4 (https://github.com/hybsearch/clustalo). Gene models were plotted with the R package gggenes (https://github.com/wilkox/gggenes).

## Results and discussion

### Sequencing and assembly

We generated 10.5 billion base pairs of nanopore sequencing data: a total of 1,543,039 reads with a read N50 of 13,472 (Supplementary Table 1). A draft assembly was generated with HiFiasm using reads longer than 5,000 base pairs, which produced a 209.0 Mbp assembly contained in 224 contigs. A combination of coverage, GC%, and blast hits from BlobTools and FCS results were used to identify nonnuclear contigs and curate the assembly. After scaffolding, purging duplicate haplotigs, removing 1 misassembled mitochondrial contig (Supplementary Table 2), and extracting the *Wolbachia w*Tkk and mitochondrial genomes, the final assembly was 205.2 Mbp in 154 contigs, with 99.13% of distinct k-mers recovered and an average of 34× coverage (Table 2). The *T. kaykai* assembly falls in the middle of the size range for the genus, 187.6 Mbp in *T. pretiosum* to 235.4 Mbp in one of the *T. brassicae* (Table 1). Additionally, this size closely aligns with a flow cytometry-based estimate of 216 Mbp for a different colony of *T. kaykai*, “LC19-1” (van Vugt *et al*. 2005). The GC% of *Trichogramma* genomes appears to be highly conserved, with all at 40%.

**Table 2.** *Trichogramma kaykai* genome assembly statistics.

Metric	Draft	Final
Contigs	224	154
Total length (bp)	208,973,237	205,183,918
Min contig length	5,287	11,361
Average contig length	932,916	1,332,363
Max contig length	6,625,044	6,625,044
N50	1,906,969	2,221,389
L50	34	31
%GC	39.60	39.63
K-mer recovery^b^	99.92	99.13
Compleasm^a^	92.69% [S: 91.47%, D: 1.22%] F: 0.82%, M: 6.49%, *n* = 5991	92.67% [S:91.74%, D: 0.93%] F: 0.82%, M:6.51%, *n* = 5991

^a^Standard BUSCO annotation: Complete BUSCOs (C) [Complete and single-copy BUSCOs (S), Complete and duplicated BUSCOs (D)], Fragmented BUSCOs (F), Missing BUSCOs (M), Total BUSCO groups searched (*n*). Hymenoptera dataset used for determining completeness. The final compleasm metric is the same as what is reported for *T. kaykai* in Table 1.

^b^As per Merqury (Rhie *et al*. 2020) results.

Quality assessments indicate that the genome assembly is quite complete, with 92.67% of Hymenopteran BUSCO loci present as complete coding sequences (Table 2). These metrics are on par with other well-assembled *Trichogramma* genomes (Table 1). Comparative genomics of *T. pretiosum* relative to other hymenopterans indicated that these wasps have undergone a large number of core gene losses and have highly accelerated rates of protein evolution (Lindsey *et al*. 2018a), so we do not expect BUSCO scores close to 100% even for a “perfect” assembly. Notably, *Trichogramma* genome assemblies with BUSCO scores >96% have been reported (Ferguson *et al*. 2020). However, and for clarity, we note that these are based on the Insecta BUSCO dataset rather than the Hymenoptera dataset (Table 1).

### Genome methylation

We determined 5′ methylation at cytosines in a CpG context based on the direct sequencing basecalls. Less than 1% of CpGs were methylated: 0.67% of CpGs had 5mC (methyl) modifications and 0.18% had 5hmC (hydroxymethyl) modifications. While this is a low level of methylation as compared to vertebrates, this is not atypical for insects (Hunt *et al*. 2013). Importantly, this level of methylation closely mirrors the number of methylated CpG sites identified in *T. pretiosum* using bisulfite sequencing (Lindsey *et al*. 2018a; Wu *et al*. 2020).

### Analysis of repetitive DNA

**Fig. 2.**
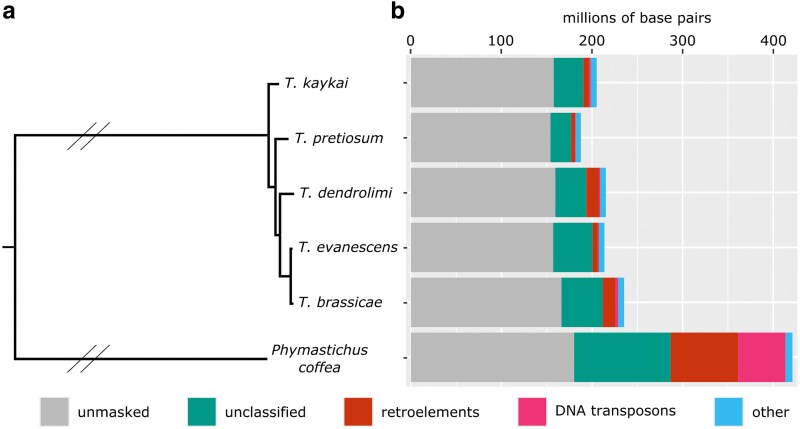
Comparative genomics of *Trichogramma.* a) Whole genome phylogeny of 5 *Trichogramma* species and outgroup *P. coffea* (Hymenoptera: Eulophidae). Double slashes indicate branches that were shortened to half their length for ease of visualization. b) Repetitive content of each genome, corresponding to the taxa in a). “Other” includes rolling circles, simple repeats, and low complexity repeats.

**Fig. 3.**
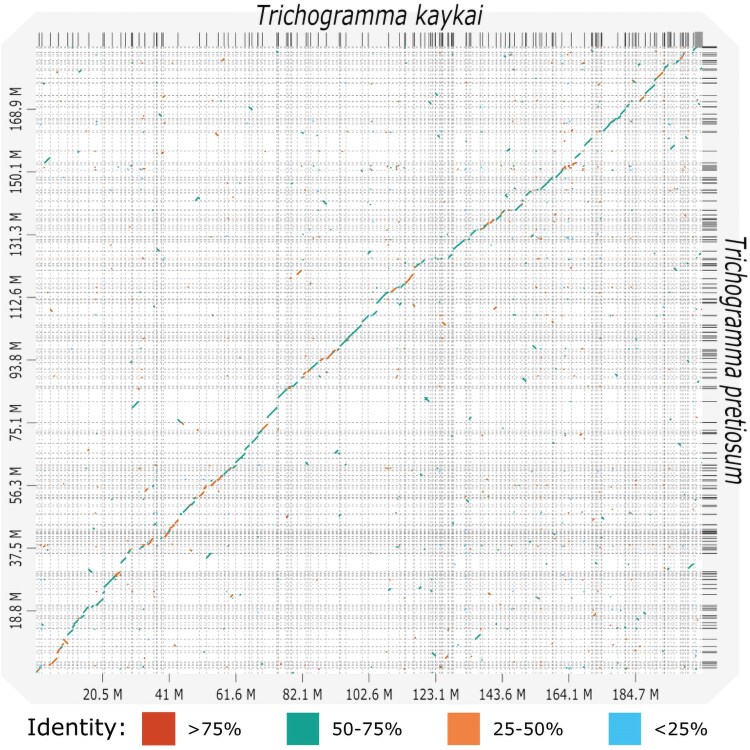
Synteny is highly conserved between *T. kaykai* and *T. pretiosum*. Dot plot indicating syntenic regions between *Trichogramma* genomes. Dots are colored according to percent identity.

*Trichogramma kaykai* is sister to all other *Trichogramma* species with published genomes (Fig. 2a). Across the genus, repetitive content appears to be relatively conserved. Repetitive sequences account for between 17.9% and 29.39% of the total genome lengths (Fig. 2b, Supplementary Table 3). This is in contrast to the outgroup species, *P. coffea* (Hymenoptera: Eulophidae), that has a 421 Mbp genome with more than half (57.21%) attributed to repetitive sequences (Fig. 2b, Supplementary Table 3). Across *Trichogramma*, the majority of repetitive sequences are unclassified. In *T. kaykai*, 3% of the genome is derived from retroelements, <1% from DNA transposons, and around 3% of the genome is simple and low complexity repeats (Table 3). We then assessed the level of synteny between *T. kaykai* and *T. pretiosum* by cross-mapping similar genomic sequences with D-GENIES (Fig. 3). A large proportion (60.34%) of the *T. kayaki* genome shares 50–75% identity with *T. pretiosum*, and there are high levels of synteny across the 2 assemblies (Fig. 3).

**Table 3.** Interspersed repeats in *T. kaykai.*

Name	Number	Length (bp)	Percent (%)
Retroelements	8,065	6,578,491	3.21
Penelope class	178	64,474	0.03
LINE class	4,347	2,948,008	1.44
L2/CR1/Rex	794	346,909	0.17
R1/LOA/Jockey	2,662	1,949,224	0.95
R2/R4/NeSL	291	358,147	0.17
LTR class	3,708	3,630,483	1.77
BEL/Pao	244	323,844	0.16
Ty1/Copia	376	345,668	0.17
Gypsy/DIRS1	3,088	2,960.971	1.44
DNA transposons	5,109	1,833,021	0.89
hobo-Activator	274	65,946	0.03
Tc1-IS630-Pogo	1310	259,829	0.13
Rolling-circles	653	370,241	0.18
Unclassified	112,931	32,542,167	15.86
Total interspersed repeats		40,953,679	19.96
Simple repeats	148,252	5,475,019	2.67
Low complexity	15,358	694,790	0.34
Bases masked		47,493,729	23.15

### Genome annotation

We annotated the *T. kaykai* genome and identified 19,689 genes (Table 4). These genes corresponded to 21,994 transcripts, with a mean of 4.6 exons per mRNA (Table 4). Compared with other *Trichogramma* species, this is a larger number of annotated genes (e.g. 13,395 in *T. pretiosum*, 16,905 in *T. brassicae*). However, our same annotation pipeline annotated 18,784 genes (with 21,249 transcripts) in *T. pretiosum*, so we hypothesize that most of the variation is due to the gene evidence and annotation pipelines.

**Table 4.** Annotation metrics for the *T. kaykai* genome.

Metric	Value^a^
Number of genes	19,689
Number of mRNAs	21,994
Number of exons	101,158
Number of introns	79,164
Mean exons per mRNA	4.6
Total gene length	61,670,814 bp
Longest gene	101,398 bp
Mean gene length	3,132 bp
Longest CDS	54,792 bp
Mean CDS length	1,440 bp
Longest exon	14,940 bp
Mean exon length	314 bp

^a^Base pairs = bp.

### Mitogenome

**Fig. 4.**
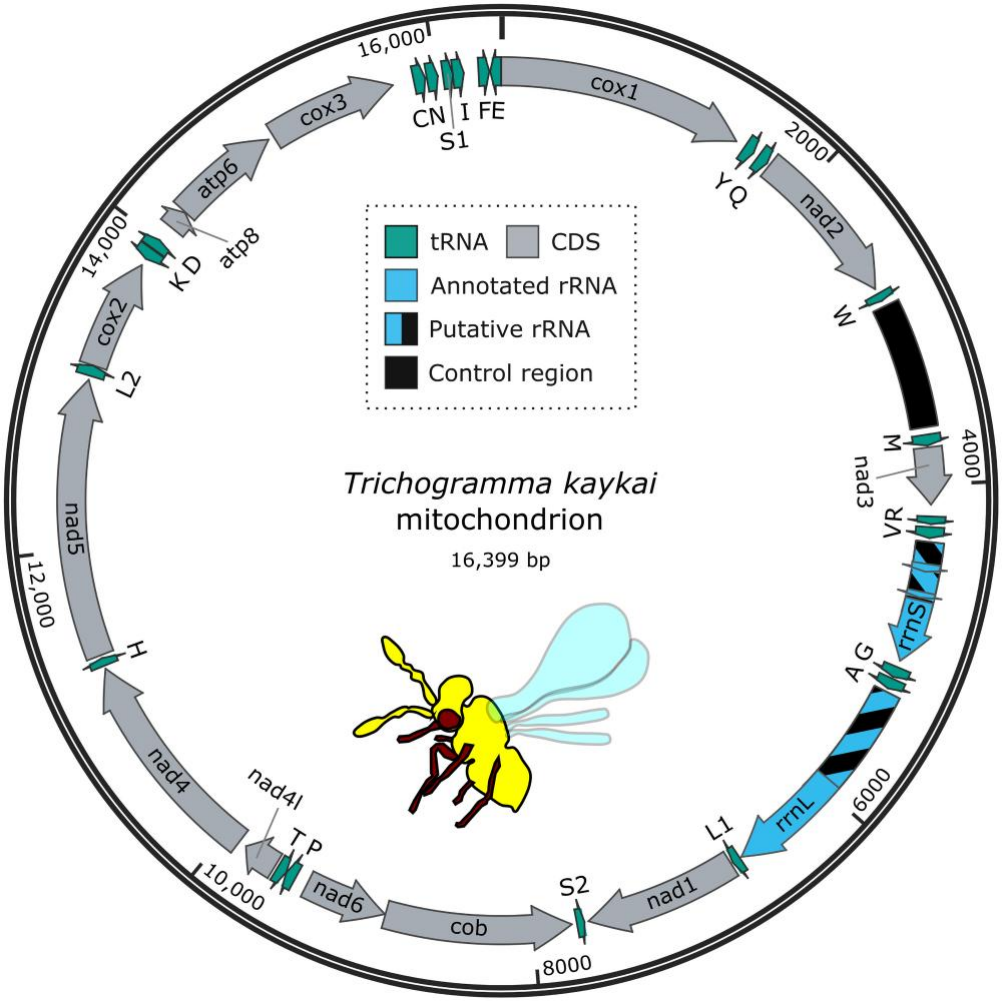
Mitochondrial genome of *T. kaykai*. Genes were annotated with MITOS2 (Bernt *et al*. 2013). Putative regions of rRNAs that were not correctly annotated by MITOS2 are indicated with stripes. The control region and the putative full length rRNAs were identified based on homology and gene order of other *Trichogramma* mitochondria (Chen *et al*. 2018). Transfer RNAs are denoted by IPUC-IUB amino acid codes.

We identified the mitogenome based on GC content (14.81%), size (16,399 bp), and coverage (3708×). Annotation revealed all expected mitochondrial tRNAs and coding genes (Fig. 4). MITOS2 annotated a single large rRNA of only 712 bp and 3 regions (387, 49, and 38 bp) as small rRNAs. Comparison to other Trichogrammatid mitochondrial genomes indicated that the large rRNA annotation had been truncated on the 5′ end, and the small rRNA annotation had been fragmented (Fig. 4), which is likely due to the high level of divergence and rearrangement in these mitochondrial genomes as compared to those that makeup the reference database (Chen *et al*. 2018; Donath *et al*. 2019). A 878 bp region between the tRNAs for tryptophan (W) and methionine (M) corresponds to the putative control region identified in other Trichogrammatid mitochondrial genomes (Chen *et al*. 2018).

### PI *Wolbachia* strain *w*Tkk

**Fig. 5.**
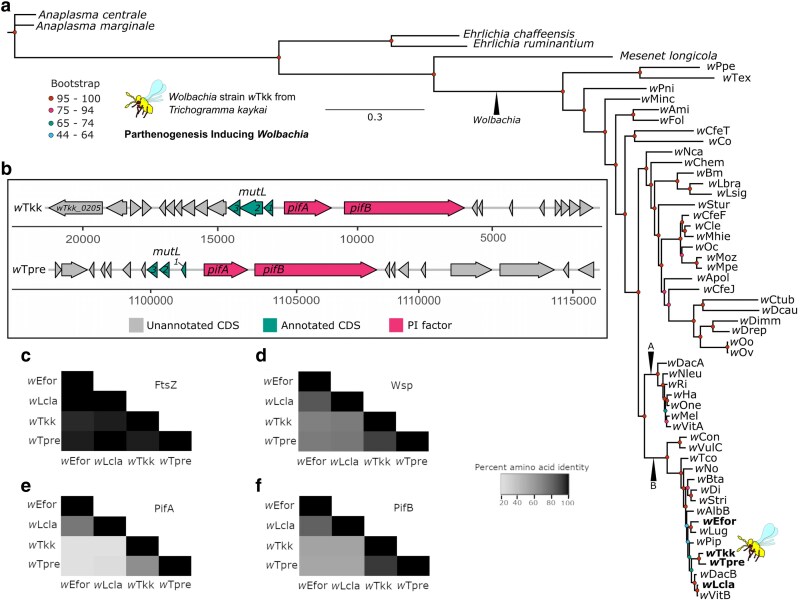
PI *Wolbachia* strain *w*Tkk. a) Maximum likelihood-based phylogeny of *Wolbachia* strains and Rickettsiales outgroups based on 78 core, single-copy, protein coding genes (a total of 30,477 aligned amino acid sites). b) Gene models for a predicted remnant prophage region that contains the parthenogenesis factors *pifA* and *pifB*, in *w*Tkk and *w*Tpre. c–f) *Wolbachia* protein divergence. Percent identity of c) FitsZ, d) Wsp, e) PifA, and f) PifB. Parthenogenesis inducing (PI) factor (*pif*), coding sequence (CDS).

We assembled a near-complete *Wolbachia* genome of the *w*Tkk strain: ∼1.12 Mbp contained in 4 contigs, sequenced at 55× coverage (Table 5). Phylogenetic reconstruction revealed that *w*Tkk is in the “Supergroup B” clade of *Wolbachia*, and is sister to *w*Tpre, which infects *T. pretiosum* (Lindsey *et al*. 2016) (Fig. 5a). The *w*Tkk and *w*Tpre genomes are similar in size: the *w*Tpre assembly (a single scaffold) is just slightly larger at 1,133,709 bp (Lindsey *et al*. 2016). Also in Supergroup B are the PI strains *w*Lcla [which infects the parasitoid wasp *Leptopilina clavipes* (Hymenoptera: Figitidae)] (Pannebakker *et al*. 2004 ) and *w*Efor, [which infects the parasitoid wasp *Encarsia formosa* (Hymenoptera: Aphelinidae)] (Zchori-Fein *et al*. 1992).

**Table 5.** *Wolbachia* strain *w*Tkk genome assembly and annotation.

Metric	*w*Tkk
Contigs	4
Length (bp)	1,119,794
%GC	33%
Compleasm^a^	93.96% [S: 93.96%, D: 0%] F: 0.55%, M: 5.49%, *n* = 364
CDS	1,265
rRNAs	3
tRNAs	34

^a^Standard BUSCO annotation: Complete BUSCOs (C) [Complete and single-copy BUSCOs (S), Complete and duplicated BUSCOs (D)], Fragmented BUSCOs (F), Missing BUSCOs (M), Total BUSCO groups searched (*n*). Rickettsiales dataset used for determining completeness.

We next queried the PI *Wolbachia* genomes for the recently identified PI factors, *pifA* and *pifB* (Fricke and Lindsey 2024). These PI genes, *pifA* and *pifB* were originally identified in *w*Tpre and *w*Lcla (Fricke and Lindsey 2024), and a *pifA* homolog has since been identified in *w*Efor (there named “*piff*”) (Li *et al*. 2024). We identified a single copy of each *pif* in the *w*Tkk genome, encoded next to each other within a remnant prophage region (Fig. 5b), as is typical of many other *Wolbachia* loci that induce host reproductive manipulations (Bordenstein and Bordenstein 2016; LePage *et al*. 2017; Lindsey *et al*. 2018b; Shropshire *et al*. 2018; Perlmutter *et al*. 2019; Fricke and Lindsey 2024). In both *w*Tpre and *w*Tkk, the immediate *pif-*regions are syntenic. In both strains, upstream of *pifA*, 3 tandem CDS were annotated as *mutL*: a pseudogenization due to nonsense mutations and fragmentation of the coding region into multiple open reading frames (Fig. 5b), a previously characterized feature of *w*Tpre genome evolution (Lindsey *et al*. 2016). Finally, we also identified a *pifB* homolog in *w*Efor (corresponding to GenBank Accession WP_343288993.1), encoded approximately 5 kb from *pifA*/*piff* (WP_343288992.1).

To understand the evolution of PI proteins, we compared the divergence of the Pifs to a slowly evolving core bacterial gene, FtsZ (Fig. 5c) (Baldo *et al*. 2006), and a rapidly evolving *Wolbachia*-specific outer membrane protein, *Wolbachia* Surface Protein (Wsp, Fig. 5d) (Baldo *et al*. 2010). The *Trichogramma*-infecting strains, *w*Tpre and *w*Tkk were 93% similar at PifB, which is only slightly more conserved than the fast evolving-Wsp (91%). In contrast, the PifB proteins of *w*Tpre and *w*Tkk were 53–55% similar to the more distantly related *w*Efor and *w*Lcla homologs, as compared to 70–73% similarity at Wsp (Fig. 5f). PifA appears to be especially rapidly evolving: there was 65% similarity between the PifA of *Trichogramma*-infecting strains, and only 21–23% similarity between the *Trichogramma*-infecting and non-*Trichogramma*-infecting strains (Fig. 5e).

## Summary

We report here a high-quality assembly for the parasitoid wasp *T. kaykai* along with genomes for its mitochondrion and associated PI *Wolbachia* strain, *w*Tkk. At the time of our analyses, 5 other *Trichogramma* genomes were available on NBCI: one each from the *Trichogramma* species *pretiosum*, *dendrolimi*, and *evanescens*, and 2 assemblies for *T. brassicae*. These species are some of the more commonly available *Trichogramma* sold as biological control agents of lepidopteran pests (Knutson 1998; Cherif *et al*. 2021). To date, all *Trichogramma* species assayed for karyotype have a haploid genome of 5 chromosomes (2*n* = 10) (Gokhman and Quicke 1995; Van Vugt *et al*. 2009; Gokhman *et al*. 2017; Farsi *et al*. 2020; Gokhman 2020). While chromosome number and approximate genome size are conserved, there do appear to be species-specific differences in chromosome morphometrics (e.g. centromere location, arm lengths, chromosome sizes) (Gokhman *et al*. 2017; Farsi *et al*. 2020; Gokhman 2020). Nevertheless, our analyses indicate that a high level of synteny has been conserved within the genus (Fig. 3).

Of the *Trichogramma* genome sequencing efforts, 1 other reports a *Wolbachia* genome: strain *w*Tpre, from *T. pretiosum* (Lindsey *et al*. 2016). The 2 *Trichogramma*-infecting strains*, w*Tpre and *w*Tkk, are closely related members of the “Supergroup B” clade, which contains a suite of other arthropod-infecting strains, including other PI from a range of host insects (Lindsey *et al*. 2016; Scholz *et al*. 2020). While *Wolbachia* are maternally transmitted, across longer evolutionary time scales there is a significant amount of horizontal transfer, and often sister strains infect distantly related hosts (Bailly-Bechet *et al*. 2017; Scholz *et al*. 2020). However, the PI-*Wolbachia* infecting *Trichogramma* appear to have a single origin (Schilthuizen and Stouthamer 1997; Poorjavad *et al*. 2012; Almeida and Stouthamer 2018). In line with this, our phylogenetic analyses recover a sister relationship of *w*Tpre and *w*Tkk (Fig. 5a). These PI-*Wolbachia* still undergo host switching within *Trichogramma sp.* (i.e. there is no co-cladogenesis) (Huigens *et al*. 2000, 2004; Almeida and Stouthamer 2018), but the fact that this clade of *Wolbachia* seem restricted to a single host genus makes them an interesting case study for host adaptation and the evolution of their PI effector proteins. Indeed, comparisons of Pifs across PI strains indicate a rapid rate of evolution that may reflect adaptation to specific hosts. PifA appears to be especially rapidly evolving, which we hypothesize is due to its role in directly interfacing with the host sex determination system, also rapidly evolving (Blackmon *et al*. 2017; Fricke and Lindsey 2024; Li *et al*. 2024).

In addition to the PI-*Wolbachia* present in *T. kaykai*, the PSR chromosome found in some males offers another opportunity to understand the evolution of sex ratio distortion (Zhang and Ferree 2024). Another such PSR chromosome has been described in the parasitoid wasp *Nasonia vitripennis* (Hymenoptera: Pteromalidae) (Nur *et al*. 1988; Werren 1991), and there is a probable PSR chromosome in *T. dendrolimi* (Liu *et al*. 2019). The PSR chromosomes from *Nasonia* and *T. kaykai* have independent origins, albeit a very similar paternal genome elimination phenotype (van Vugt *et al*. 2003; Zhang and Ferree 2024). Curiously, both PSR chromosomes seem to have originated from hybridization events in which chromosomal regions with abundant repetitive elements were transferred in via a close relative (McAllister and Werren 1997; van Vugt *et al*. 2005; Van Vugt *et al*. 2009). In contrast to *T. kaykai*, *Nasonia* are not known to host any PI symbionts (Beukeboom and Van De Zande 2010). However, some *N. vitripennis* do host male-killing bacteria: *Arsenophonus nasoniae* (Gherna *et al*. 1991; Ferree *et al*. 2008). The PSR chromosomes are likely playing a key role in male-rescue, which balances the male-eliminating cytoplasmic factors in both systems (either elimination by conversion to female via PI-*Wolbachia* in *Trichogramma*, or, elimination via death via *Arsenophonus* in *Nasonia*). The *Wolbachia*-infected line of *T. kaykai* reported here will enable the long-term maintenance of PSR chromosomes in the lab, and in the future, we hope to re-collect PSR-containing males from the native range to better understand the evolution of these selfish genetic elements.

## Data Availability

This Whole Genome Shotgun project has been deposited at DDBJ/ENA/GenBank under the BioProject accession PRJNA1150630. BioSample accessions for *T. kaykai* and *Wolbachia* strain *w*Tkk are SAMN43292057 and SAMN43292058, respectively. Sequencing reads are deposited under SRR30339640. The Whole Genome Shotgun project for the *T. kaykai* nuclear genome has been deposited at DDBJ/ENA/GenBank under the accession JBJJXI000000000; the version described in this paper is version JBJJXI020000000. The Whole Genome Shotgun project for the *Wolbachia* strain *w*Tkk genome has been deposited at DDBJ/ENA/GenBank under the accession JBJJXK000000000; the version described in this paper is version JBJJXK010000000. The *T. kaykai* mitochondrial genome is available under accession number PQ667799. Supplementary materials are available on the GSA Figshare portal (https://doi.org/10.25387/g3.29226122) and include: (A) Supplementary Table 1. Nanopore sequencing statistics, (B) Supplementary Table 2. Details on draft assembly curation, (C) Supplementary Table 3. Comparison of interspersed repeats between *T. kaykai* and related species, (D) Supplementary File 1. *Trichogramma kaykai g*enome annotations in GFF3 format, and (E) Supplementary File 2. Analysis notebook with bioinformatics workflows and scripts. A voucher of the *T. kaykai* KSX58 colony is available at the University of California Riverside Insect Collection: UCRC_ENT00496298.
